# AAV9-mediated *SMN* gene therapy rescues cardiac desmin but not lamin A/C and elastin dysregulation in *Smn*^*2B/−*^ spinal muscular atrophy mice

**DOI:** 10.1093/hmg/ddad121

**Published:** 2023-07-27

**Authors:** Sharon J Brown, Darija Šoltić, Silvia A Synowsky, Sally L Shirran, Ellie Chilcott, Hannah K Shorrock, Thomas H Gillingwater, Rafael J Yáñez-Muñoz, Bernard Schneider, Melissa Bowerman, Heidi R Fuller

**Affiliations:** School of Pharmacy and Bioengineering, Keele University, Keele ST5 5BG, UK; Wolfson Centre for Inherited Neuromuscular Disease, TORCH Building, RJAH Orthopaedic Hospital, Oswestry SY10 7AG, UK; School of Pharmacy and Bioengineering, Keele University, Keele ST5 5BG, UK; Wolfson Centre for Inherited Neuromuscular Disease, TORCH Building, RJAH Orthopaedic Hospital, Oswestry SY10 7AG, UK; BSRC Mass Spectrometry and Proteomics Facility, University of St Andrews, St Andrews KY16 9ST, UK; BSRC Mass Spectrometry and Proteomics Facility, University of St Andrews, St Andrews KY16 9ST, UK; AGCTlab.org, Centre of Gene and Cell Therapy, Centre for Biomedical Sciences, Department of Biological Sciences, School of Life Sciences and the Environment, Royal Holloway University of London, Egham Hill, Egham, Surrey TW20 0EX, UK; Edinburgh Medical School: Biomedical Sciences, Euan MacDonald Centre for Motor Neurone Disease Research, University of Edinburgh, Edinburgh EH8 9XD, UK; Edinburgh Medical School: Biomedical Sciences, Euan MacDonald Centre for Motor Neurone Disease Research, University of Edinburgh, Edinburgh EH8 9XD, UK; AGCTlab.org, Centre of Gene and Cell Therapy, Centre for Biomedical Sciences, Department of Biological Sciences, School of Life Sciences and the Environment, Royal Holloway University of London, Egham Hill, Egham, Surrey TW20 0EX, UK; Bertarelli Platform for Gene Therapy, Ecole Polytechnique Fédérale de Lausanne (EPFL), 1202 Geneva, Switzerland; Brain Mind Institute, Ecole Polytechnique Fédérale de Lausanne (EPFL), 1015 Lausanne, Switzerland; Wolfson Centre for Inherited Neuromuscular Disease, TORCH Building, RJAH Orthopaedic Hospital, Oswestry SY10 7AG, UK; School of Medicine, Keele University, Keele ST5 5BG, UK; School of Pharmacy and Bioengineering, Keele University, Keele ST5 5BG, UK; Wolfson Centre for Inherited Neuromuscular Disease, TORCH Building, RJAH Orthopaedic Hospital, Oswestry SY10 7AG, UK

## Abstract

Structural, functional and molecular cardiac defects have been reported in spinal muscular atrophy (SMA) patients and mouse models. Previous quantitative proteomics analyses demonstrated widespread molecular defects in the severe Taiwanese SMA mouse model. Whether such changes are conserved across different mouse models, including less severe forms of the disease, has yet to be established. Here, using the same high-resolution proteomics approach in the less-severe *Smn^2B/−^* SMA mouse model, 277 proteins were found to be differentially abundant at a symptomatic timepoint (post-natal day (P) 18), 50 of which were similarly dysregulated in severe Taiwanese SMA mice. Bioinformatics analysis linked many of the differentially abundant proteins to cardiovascular development and function, with intermediate filaments highlighted as an enriched cellular compartment in both datasets. Lamin A/C was increased in the cardiac tissue, whereas another intermediate filament protein, desmin, was reduced. The extracellular matrix (ECM) protein, elastin, was also robustly decreased in the heart of *Smn^2B/−^* mice. AAV9-*SMN1*-mediated gene therapy rectified low levels of survival motor neuron protein and restored desmin levels in heart tissues of *Smn^2B/−^* mice. In contrast, AAV9-*SMN1* therapy failed to correct lamin A/C or elastin levels. Intermediate filament proteins and the ECM have key roles in cardiac function and their dysregulation may explain cardiac impairment in SMA, especially since mutations in genes encoding these proteins cause other diseases with cardiac aberration. Cardiac pathology may need to be considered in the long-term care of SMA patients, as it is unclear whether currently available treatments can fully rescue peripheral pathology in SMA.

## Introduction

Spinal muscular atrophy (SMA) arises from insufficient levels of survival motor neuron (SMN) protein due to a homozygous mutation/deletion in the *SMN1* gene (1). Although humans possess the *SMN2* gene (2), this generates only a limited amount of functional SMN (~10%) with the majority (~90%) being an unstable, truncated form of the protein (3). SMA patients present with a range of clinical phenotypes with varying severity, which generally, but not always, have an inverse relationship with *SMN2* copy number (4). Recent advances in SMA treatments aim to increase the levels of full-length SMN by either upregulating the *SMN2* gene or by delivering the *SMN1* gene directly to the cells via a viral vector (5). Neither treatment option is fully effective and evidence is emerging of new patient phenotypes with long-term treatment outcomes remaining unknown (5).

Although SMA is typically considered a motor neuron disease, the ubiquitous nature of SMN production has prompted research into the effects of reduced levels of SMN in peripheral tissues and organs, in both patients and animal models of SMA. Evidence demonstrating that SMA is a multisystem disease has been accruing (6,7) and impairment of cardiac function in mouse models of SMA and in SMA patients described (8–15). Examples of cardiac dysfunction include observations of bradycardia, dilated cardiomyopathy and decreased contractility in SMNΔ7 mice (12), and electrocardiogram abnormalities and thickened myocardium in patients with milder forms of SMA (9,16), whereas a retrospective study of patients with SMA Type I identified ~ 24% of patients with severe symptomatic bradycardia (10). Congenital heart defects, such as septal defects, in severe Type I patients (11) and in the severe Taiwanese SMA mouse model (14) have also been noted. A study in which the incidence of health insurance claims in Type I–III patients pre-SMA diagnosis were compared with control patients found valve disorders, cardiomyopathies, septal defects and premature beats to be increased (17). Furthermore, a systematic review of more than 70 studies in which 264 SMA patients were identified with cardiac pathology found a tendency for structural abnormalities to occur in the more severe form of SMA (Type I), whereas in patients with less severe SMA, cardiac rhythm disturbances were more common (18).

SMN protein is found in both the cytoplasm and nucleus of cells, and typically associated with structures called Gemini of the coiled bodies (Gems) in the nucleus. There is also evidence that SMN localizes to structural components such as the sarcomere in striated muscle fibres (19) and the *Z*-disc in mice cardiac myofibrils (20), but its function in cardiac muscle remains unknown. Most studies have focussed on the more severe form of SMA, including our previous quantitative proteomics study in which we demonstrated widespread molecular defects in heart tissue from the severe Taiwanese SMA mouse model compared with healthy controls (15). In particular, a robust increase in the intermediate filament protein, lamin A/C, was observed, which we hypothesized may contribute to the impairment of cardiac function by causing nuclear stiffness in the cardiomyocytes.

A recent study that focussed on the FoxO family of transcription factors in heart tissues from the less-severe *Smn^2B/−^* SMA mouse model, found no significant pathology related to these factors or their downstream targets of proteosomal and autophagosomal degradation (21), but the full extent of molecular consequences and their implications remain unknown for this less-severe SMA mouse model. A recent transcriptome study of cardiomyocytes isolated from a severe SMA mouse model and cardiomyocytes generated from induced pluripotent stem cells (iPSCs) derived from an SMA Type II patient, however, found that SMN deficiency disrupts muscle cell and fibre development, muscle function and Ca^2+^ handling (22). Cell studies are extremely useful, but cell isolation and subsequent culture changes the physiological stresses that they are exposed to in comparison to those experienced *in vivo*. Thus, we undertook a quantitative proteomic comparison of heart tissues from the less-severe *Smn^2B/−^* SMA mouse model and age-matched wild-type (WT) mice and compared these findings with those identified in our previous study of heart tissues from the severe Taiwanese SMA mouse model (15).

We report evidence of widespread protein dysregulation in *Smn^2B/−^* mouse hearts compared with age-matched WT mice, some of which was common to the Taiwanese SMA mouse model. In agreement with our previous findings from the Taiwanese model (15), the intermediate filament protein, lamin A/C, was increased in expression in hearts from the less-severe *Smn^2B/−^* SMA mouse model, whereas desmin, another intermediate filament protein, was found to be decreased in heart tissues in both mouse models. The extracellular matrix (ECM) protein, elastin, was also significantly decreased in the heart tissues of the *Smn^2B/−^* mouse. Whilst AAV9-mediated *SMN1* delivery restored desmin levels in the *Smn^2B/−^* SMA mouse model to those seen in WT mice, this treatment did not rescue the increased levels of lamin A/C or the decreased levels of elastin, even though levels of SMN were significantly enhanced when a codon-optimized transgene was administered. Together these findings suggest that post-natal AAV9-mediated *SMN1* delivery may not rectify all the dysregulated proteins found in the *Smn^2B/−^* mouse model of SMA, and as these proteins have the potential to detrimentally impact heart function, additional cardiac monitoring may prove useful in the long-term care of SMA patients.

## Results

### Quantitative proteomics analysis of heart tissue from a mouse model of less-severe SMA reveals widespread molecular defects

To determine the molecular consequences of SMN depletion in heart tissue from the less-severe *Smn^2B/−^* SMA mouse model, a relative quantitation proteomic comparison against age-matched WT mouse hearts was undertaken using iTRAQ™ mass spectrometry analysis at the symptomatic time-point of P18. This approach identified 3105 proteins in total (Supplementary Material, Table S1) after removing proteins identified from only one peptide and those which matched to the decoy search (i.e. prefixed with ‘REVERSED…’). Of these, 2479 were identified with a 5% local false-discovery rate. From these, 277 proteins met the specified criteria (an unused ProtScore (conf) > 0.05, detected by ≥2 unique peptides, and a statistically significant (*p* ≤ 0.05) fold change of ≥ 1.25 or ≤ 0.8) for differential expression in the *Smn^2B/−^* mouse heart compared with age-matched WT hearts, of which 156 proteins were increased and 121 were decreased in expression in SMA mouse hearts (Supplementary Material, Table S2).

**Figure 1:**
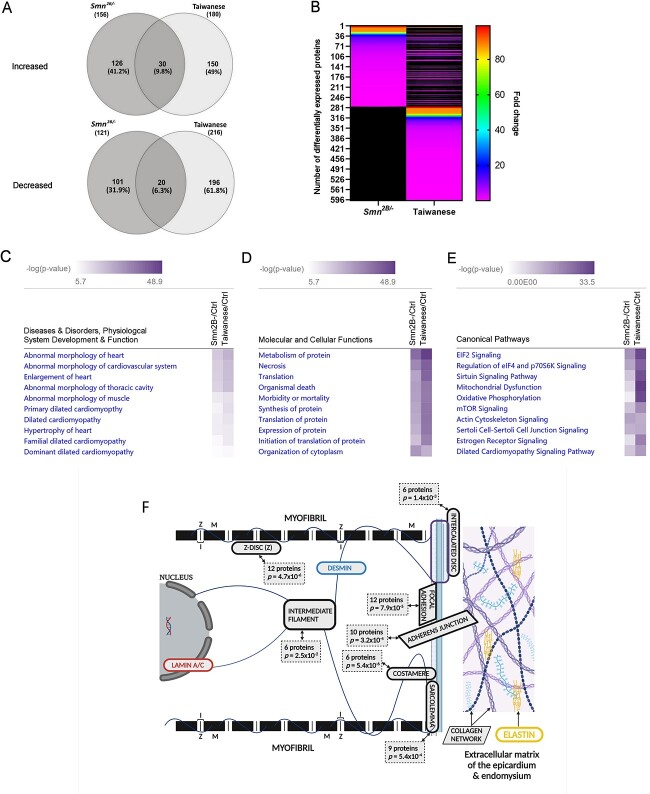
Summary and bioinformatics analysis of the proteomic profiles of heart tissues differentially abundant in the *Smn^2B/−^* and Taiwanese mice models of SMA compared with age-matched WT mice. (**A**) Venn diagram showing an overlap of 50 proteins identified as significantly up- or down-regulated in both *Smn^2B/−^* and Taiwanese mouse models of SMA (an unused ProtScore (conf) > 0.05, detected by ≥2 unique peptides, and a statistically significant (*p* ≤ 0.05) fold change of ≥ 1.25 or ≤ 0.8). (**B**) Heat map illustrating the size of fold-change in proteins found to be dysregulated in both mice models of SMA. Two-way comparisons generated in IPA® in which the following were ranked by –log(*p*-value) for each SMA mouse model: (**C**) disease and disorders, physiological system development and function; (**D**) molecular and cellular functions; and (**E**) canonical pathways. (**F**) Schematic illustrating the cellular components impacted by the significantly dysregulated proteins of the *Smn^2B/−^* mouse model of SMA as determined following DAVID analysis. Image created with BioRender.com.

To guide the direction of further studies, we were interested to understand whether any of the differentially abundant proteins identified in heart tissue from the less-severe *Smn^2B/−^* SMA mouse model were conserved with those identified previously from the severe Taiwanese SMA mouse model (15). The iTRAQ data pertaining to the Taiwanese model were derived in the same experiment as the *Smn^2B/−^* data reported here, but only the Taiwanese section of the data was published previously (15). Compared with their respective age-matched WT mice (i.e. P18 for the *Smn^2B/−^* & P8 for the Taiwanese), and using the same criteria (*p* ≤ 0.05, fold change of ≥1.25 or ≤ 0.8, > 1 unique peptide), 50 proteins were found to be commonly dysregulated in both mouse models of SMA (Supplementary Material, Table S3). Of these, 30 were up-regulated and 20 down-regulated with statistical significance in both models (Fig. 1A) with a similar fold-change in most dysregulated proteins (Fig. 1B).

The list of differentially abundant proteins from both mouse models was also compared using the comparison analysis function of the curated bioinformatics platform, Ingenuity Pathway Analysis (IPA®). This two-way comparison in which the enriched terms were ranked by absolute *p*-value, demonstrated a similar enrichment of terms for ‘diseases & disorders, physiological system development & function’ after removal of cancer related terms. In particular, ‘abnormal morphology’ and ‘dilated cardiomyopathy’ were highly enriched in both mouse models (*Smn^2B/−^* mouse model, *p*-value range 3.72 × 10^−19^ to 2.65 × 10^−7^; Taiwanese mouse model, *p*-value range 9.52 × 10^−24^ to 4.69 × 10^−8^) (Fig. 1C). Similarly, for ‘molecular & cellular functions’, terms relating to ‘translation, synthesis and metabolism of proteins’ and ‘cell & organismal death’ were significantly enriched among the differentially abundant proteins common to both mouse models (*Smn^2B/−^* mouse model, *p*-value range 5.01 × 10^−40^ to 2.65 × 10^−7^; Taiwanese mouse model, *p*-value range 9.52 × 10^−24^ to 4.69 × 10^−8^) (Fig. 1D). Various canonical pathways already linked to SMA such as ‘EIF2 signaling’ (23) and ‘mitochondrial dysfunction’ (24) were also enriched in the *Smn^2B/−^* and Taiwanese mouse models (Fig. 1E). In addition, the ‘dilated cardiomyopathy signaling pathway’ was significantly enriched in both models (*Smn^2B/−^* mouse model, *p* = 6.52 × 10^−11^, *z*-score = 1.043; Taiwanese mouse model, *p* = 2.72 × 10^−18^, *z*-score = −0.667).

Using the Database for Annotation, Visualization and Integrated Discovery (DAVID) bioinformatics tool, the cellular components with which the significantly dysregulated proteins in the *Smn^2B/−^* mouse model were associated was ascertained (Fig. 1F and Supplementary Material, Table S4A). Of particular interest were those cellular components pertinent to cardiomyocyte function including the *Z*-disk (12 proteins; *p* = 4.7x 10^−6^), the costamere (6 proteins; *p* = 5.4 × 10^−6^), focal adhesion (12 proteins; *p* = 7.9 × 10^−5^) and adherens junction (10 proteins; *p* = 3.2x10^−4^), the sarcolemma (9 proteins; *p* = 5.4 × 10^−4^), the intercalated disc (6 proteins; *p* = 1.4 × 10^−3^), and intermediate filaments (6 proteins; *p* = 2.5 × 10^−2^). When the 50 proteins significantly dysregulated in both the *Smn^2B/−^* and Taiwanese SMA mouse models were considered in the DAVID analysis, ‘intermediate filament’ (3 proteins; *p* = 4.8 × 10^−2^) remained an enriched cellular component term (Supplementary Material, Table S4B).

### The intermediate filament proteins, lamin A/C and desmin, are differentially abundant in heart tissue from two mouse models of SMA

The finding that intermediate filament proteins are differentially abundant in both SMA mouse models is of particular interest with regards to heart conditions. Mutations affecting both lamin and desmin (type V and type III intermediate filament proteins, respectively), commonly give rise to conditions associated with cardiomyopathy and heart failure (25). Of additional relevance is that mutations in *LMNA* -the lamin A/C gene- have been attributed to adult-onset conditions with an SMA-like phenotype (26,27). Lamin A was identified by iTRAQ as being increased in both the Taiwanese (15) and *Smn^2B/−^* SMA mouse models (Supplementary Material, Table S1) (albeit it just missed the criteria for differential expression in the *Smn^2B/−^* with *p* = 0.056). Desmin, on the other hand, was decreased in expression in both the Taiwanese (15) and *Smn^2B/−^* SMA mouse models, but only met the criteria for differential expression when peptides quantified with a 99% confidence were used to calculate the ratio, resulting in *p* = 0.049).

To gain further insights into the involvement of these intermediate filament proteins in SMA, we verified the differential expression of lamin A/C and desmin in heart tissue from the *Smn^2B/−^* and Taiwanese SMA mice. Quantitative western blotting confirmed a 1.94-fold and 2.06-fold increase in lamin A and C expression, respectively, in heart tissue extracts from *Smn^2B/−^* mice compared with age-matched WT mice (*p* = 0.014 and 0.030, respectively) (Supplementary Material, Fig. S1A). Immunohistochemistry analysis also confirmed increased lamin A immunoreactivity in heart tissues from the *Smn^2B/−^* mouse model compared with those from WT mice (1.74-fold increase, *p* = 0.007; Supplementary Material, Fig. S1B). This aligned with our previous findings (15) where lamin A/C levels were increased in heart tissue from the severe Taiwanese SMA mouse model. Immunohistochemistry analysis of *Smn^2B/−^* mouse heart sections revealed few lamin A/C positive cells in the ventricle lumen (Supplementary Material, Fig. S1C), in line with our previous observation from the Taiwanese SMA mouse model (15). Although we cannot rule out a minor contribution, this result confirms that the increased lamin A/C levels cannot be solely attributed to circulating blood cells. For reference, SMN levels in heart tissue extracts from the *Smn^2B/−^* mice were reduced to 0.08-fold (*p* = 0.007) of that found in WT mice (Supplementary Material, Fig. S1A).

Decreased desmin expression was also confirmed in both mouse models of SMA by western blotting, with levels being reduced in the *Smn^2B/−^* mice compared with age-matched (P18) WT mice to 0.73-fold (*p* = 0.033) and in the Taiwanese mice compared with age-matched (P8) WT mice to 0.76-fold (*p* = 0.007) (Supplementary Material, Fig. S2A). These findings were corroborated by immunohistochemistry analysis (Supplementary Material, Fig. S2B) with desmin immunoreactivity in *Smn^2B/−^* mice showing a decrease to 0.41-fold (*p* = 0.033) compared with WT (P18) mice and desmin staining in Taiwanese mice demonstrating a decrease to 0.26-fold (*p* = 0.019) compared with (P8) WT mice (Supplementary Material, Fig. S2C).

### A‌AV9-mediated *SMN1* delivery to increase SMN expression in the hearts of *Smn^2B/−^* SMA mice

**Figure 2:**
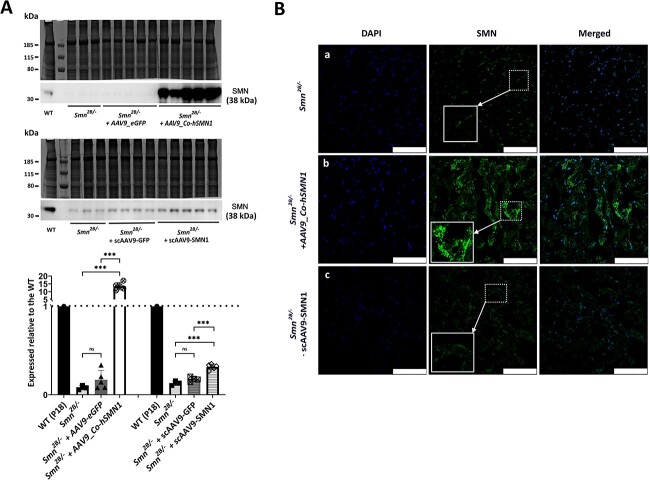
Levels and distribution of SMN in heart tissues from *Smn^2B/−^* mice following AAV9-mediated *SMN1* delivery at P0. (**A**) Representative western blots of SMN levels in heart tissues from untreated *Smn^2B/−^* mice, *Smn^2B/−^* mice following AAV9-mediated treatment with and without *SMN1* from two sources plus a corresponding age-matched WT mouse (P18). The bar graph represents average SMN levels expressed relative to the corresponding WT mouse. **(B)** Representative IMFs for SMN staining within heart tissues from untreated *Smn^2B/−^* mice and *Smn^2B/−^* mice following treatment with AAV9-mediated *SMN1* from two sources. ^*^^*^^*^p < 0.001. Scale bars represent 75 μm.

To establish the impact of currently approved gene therapy treatments, which are designed to increase SMN levels in SMA patients, on a peripheral tissue such as the heart, we examined the extent to which AAV9-mediated *SMN1* treatment restored expression levels of lamin A/C and desmin towards control mice. For these experiments, we chose to focus only on the less-severe *Smn^2B/−^* SMA model as these mice have a longer pre-symptomatic period compared with the Taiwanese mouse model allowing for a better understanding of developmental defects secondary to motor neuron loss. In addition, to validate our findings further, two distinct vectors that have previously been used in *Smn^2B/−^* mice were utilized (28,29), in particular the one vector results in enhanced expression of the *SMN1* gene (28). SMN levels were confirmed by quantitative western blotting to be significantly increased in extracts of heart tissues from *Smn^2B/−^* mice following AAV9-mediated *SMN1* replacement (Fig. 2). As expected (28), the delivery of the optimized cDNA transgene AAV9*_Co-hSMN1* resulted in enhanced levels of SMN (169-fold*; p* = 0.0002) compared with untreated *Smn^2B/−^* mice with increased levels of SMN beyond that expected in WT mice (~13.50-fold). Although the scAAV9*-SMN1-*mediated delivery also resulted in an SMN increase (2.4-fold; *p* = 0.0003) compared with untreated *Smn^2B/−^* mice, SMN levels remained below that found in WT mice (~0.32-fold) (Fig. 2A). There was no statistically significant difference in SMN expression levels between untreated *Smn^2B/−^* mice and vehicle controls (i.e. AAV9*_eGFP* (2.1-fold; *p* = 0.14); scAAV9*-GFP* (1.8-fold; *p* = 0.08) (Fig. 2A). Immunohistochemistry analysis of heart tissues from *Smn^2B/−^* mice revealed increased SMN immunoreactivity following SMN replacement, particularly in those treated with AAV9*_Co-hSMN1* (Fig. 2B).

### A‌AV9-mediated *SMN1* delivery restores desmin expression but does not correct increased lamin A/C expression in the hearts of *Smn^2B/−^* SMA mice

**Figure 3:**
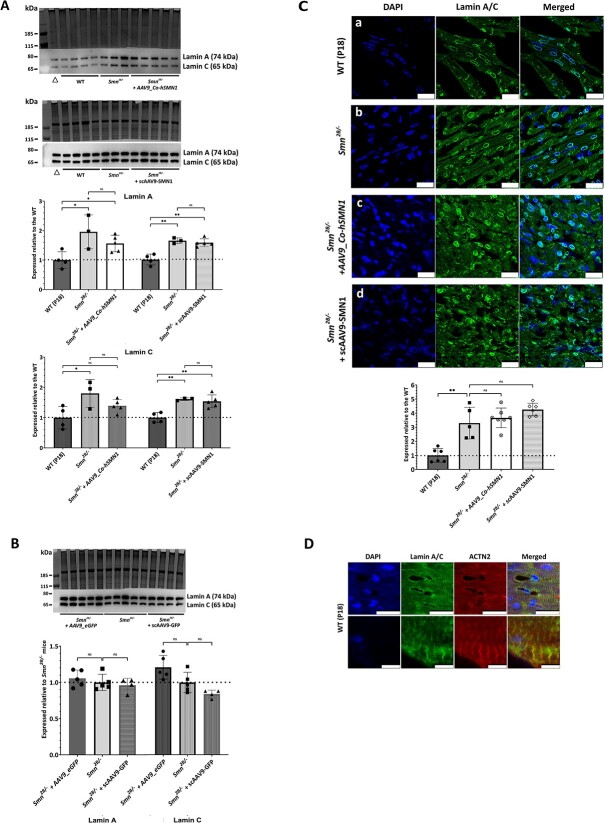
Levels and distribution of lamin A/C in heart tissues from *Smn^2B/−^* mice following AAV9-mediated *SMN1* delivery at P0. (**A**) Representative western blots of lamin A/C levels in heart tissues from WT mice (P18), untreated *Smn^2B/−^* mice and *Smn^2B/−^* mice following AAV9-mediated treatment with *SMN1* from two sources. The bar graph represents average lamin A/C levels expressed relative to WT mice. (**B**) Representative western blots of lamin A/C levels in heart tissues from untreated *Smn^2B/−^* mice and *Smn^2B/−^* mice following AAV9-mediated treatment without *SMN1* (vehicle control) from two different vector sources. The bar graph represents average lamin A/C levels expressed relative to untreated *Smn^2B/−^* mice. (**C**) Representative IMFs for lamin A/C staining within heart tissues from WT mice (P18), untreated *Smn^2B/−^* mice and *Smn^2B/−^* mice following AAV9-mediated treatment with *SMN1* from two vector sources. Corresponding bar graph reflects the area of cells stained for lamin A/C corrected for number of cells present (DAPI stain) as determined by ImageJ analysis and expressed relative to corresponding WT mice. The dashed line represents the average lamin A/C levels in WT mice and error bars represent the standard deviation from the mean. (**D**) Images demonstrating dual staining of sarcomeres with alpha-actinin 2 (red) and lamin A/C (green) in WT mice. ^*^p < 0.05; ^*^^*^p < 0.01. Scale bars represent 25 μm except the lower panel of (D) where they represent 5 μm. Δ represents a combined sample not included in the analysis.

Quantitative western blotting and immunohistochemistry analysis revealed that AAV9-mediated *SMN1* treatment did not correct the increased production of lamin A/C in *Smn^2B/−^* mouse hearts. On western blots, lamin A expression levels following delivery of AAV9*_Co-hSMN1* were 0.80-fold (*p* = 0.36) compared with untreated *Smn^2B/−^* mice and 1.56-fold (*p* = 0.04) compared with WT mice. Following scAAV9*-SMN1-*mediated delivery, lamin A levels were 0.96-fold (*p* = 0.44) compared with untreated *Smn^2B/−^* mice and 1.56-fold (*p* = 0.003) compared with WT mice (Fig. 3A). Similarly, lamin C expression levels following delivery of AAV9*_Co-hSMN1* were 0.77-fold (*p* = 0.26) compared with untreated *Smn^2B/−^* mice and 1.39-fold (*p* = 0.13) compared with WT mice and following scAAV9*-SMN1-*mediated delivery were 0.96-fold (*p* = 0.50) compared with untreated *Smn^2B/−^* mice and 1.54-fold (*p* = 0.004) compared with WT mice (Fig. 3A). There was no statistically significant difference in lamin A and C expression levels between untreated *Smn^2B/−^* mice and vehicle controls (i.e. AAV9*_eGFP* (1.06-fold (*p* = 0.46) for lamin A and 1.21-fold (*p* = 0.06) for lamin C); scAAV9*-GFP* (0.96-fold (*p* = 0.59) for lamin A and 0.84-fold (*p* = 0.06) for lamin C) (Fig. 3B).

In heart sections from WT mice, lamin A/C immunoreactivity was largely confined to the nuclear membrane, as expected, with a small proportion being localized to the sarcomere as confirmed by double-label immunohistochemistry with alpha-actinin 2, a sarcomere marker (Fig. 3C and D). In heart sections from the *Smn^2B/−^* mice, lamin A/C was significantly increased in expression at the nuclear membrane alongside a strikingly increased distribution of lamin A/C at the sarcomeres (Fig. 3C). When quantified across each section, lamin A/C immunoreactivity in heart tissues from *Smn^2B/−^* mice showed a significant increase in lamin A/C expression compared with age-matched (P18) WT mice (3.29 ± 1.13-fold; *p =* 0.004; Fig. 3C), and after treatment with AAV9*_Co-hSMN1* or scAAV9*-SMN*1 lamin A/C levels in *Smn^2B/−^* mice remained increased compared with the untreated *Smn^2B/−^* mice (3.66 ± 0.70-fold (*p* = 0.54) and 4.25 ± 0.45-fold (*p* = 0.13), respectively (Fig. 3C).

**Figure 4:**
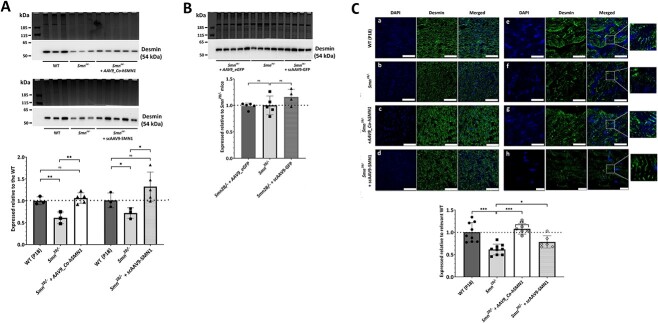
Levels and distribution of desmin in heart tissues from *Smn^2B/−^* mice following AAV9-mediated *SMN1* delivery at P0. (**A**) Representative western blots of desmin levels in heart tissues from WT mice (P18), untreated *Smn^2B/−^* mice and *Smn^2B/−^* mice following AAV9-mediated treatment with *SMN1* from two sources. The bar graph represents average desmin levels expressed relative to WT mice. (**B**) Representative western blots of desmin levels in heart tissues from untreated *Smn^2B/−^* mice and *Smn^2B/−^* mice following AAV9-mediated treatment without *SMN1* (vehicle control) from two different vector sources. The bar graph represents average desmin levels expressed relative to untreated *Smn^2B/−^* mice. (**C**) Representative IMFs for desmin staining within heart tissues from WT mice (P18), untreated *Smn^2B/−^* mice and *Smn^2B/−^* mice following AAV9-mediated treatment with *SMN1* from two vector sources. Zoomed images to highlight the reduction in desmin positive striations in untreated *Smn^2B/−^* mice in comparison with WT mice and AAV9-mediated *SMN1* treated *Smn^2B/−^* mice. Corresponding bar graph reflects the area of cells stained for desmin corrected for number of cells present (DAPI stain) as determined by ImageJ analysis and expressed relative to corresponding WT mice. The dashed line represents the average desmin levels in WT mice and error bars represent the standard deviation from the mean. ^*^p < 0.05; ^*^^*^p < 0.01; ^*^^*^^*^p < 0.001. Scale bars represent 75 μm (panels a–d), 25 μm (panels e–g) and 10 μm (panel h).

Western blot and immunohistochemistry analysis revealed that AAV9-mediated *SMN1* treatment did, however, restore the decreased expression of desmin in *Smn^2B/−^* mouse hearts towards WT levels. From western blots, desmin expression levels following delivery of AAV9*_Co-hSMN1* were 1.74-fold (*p* = 0.0095) compared with untreated *Smn^2B/−^* mice and 1.06-fold (*p* = 0.44) compared with WT mice and following scAAV9*-SMN1-*mediated delivery were 1.84-fold (*p* = 0.04) compared with untreated *Smn^2B/−^* mice and 1.31-fold (*p* = 0.13) compared with WT mice (Fig. 4A). There was no statistically significant difference in desmin expression levels between untreated *Smn^2B/−^* mice and vehicle controls (i.e. AAV9*_eGFP)* (0.99-fold (*p* = 0.88)) and scAAV9*-GFP* (1.16-fold (*p* = 0.19)) (Fig. 4B). Desmin immunoreactivity in heart sections from WT mice was typically present at the *Z*-disc (Fig. 4C). Desmin immunoreactivity in heart sections from the *Smn^2B/−^* mice was clearly reduced (Fig. 4C, high magnification images), although some striations could still be seen, the staining appearing to be variable in intensity and disorganized. Quantification of desmin immunoreactivity across each heart section from the *Smn^2B/−^* mice found desmin expression to be significantly decreased compared with age-matched (P18) WT mice (0.61 ± 0.12-fold (*p <* 0.001)) (Fig. 4C), but following treatment with AAV9*_Co-hSMN1* or scAAV9*-SMN*1 desmin levels in *Smn^2B/−^* mice were increased compared with the untreated *Smn^2B/−^* mice (1.75 ± 0.19-fold (*p <* 0.0001) and 1.28 ± 0.22-fold (*p* = 0.035)), respectively (Fig. 4C).

### Decreased elastin expression in *Smn^2B/−^* mouse heart is also refractory to AAV9-mediated *SMN1* delivery

**Figure 5:**
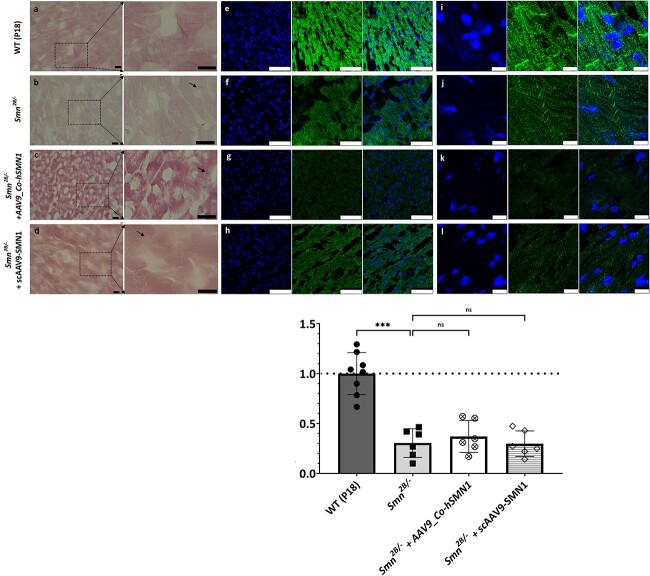
Levels and distribution of elastin in heart tissues from *Smn^2B/−^* mice following AAV9-mediated *SMN1* delivery at P0. (Panels a−d) Representative heart sections stained with Miller’s Elastin van Gieson from WT mice (P18), untreated *Smn^2B/−^* mice and *Smn^2B/−^* mice following AAV9-mediated treatment with *SMN1* from two different vector sources. Elastic fibres (blue/black); mature collagen (red); other tissues such as muscle and red blood cells (yellow). (Panels e–l) Representative IMFs for elastin staining within heart tissues from WT mice (P18), untreated *Smn^2B/−^* mice and *Smn^2B/−^* mice following AAV9-mediated treatment with *SMN1* from two vector sources. Corresponding bar graph reflects the area of cells stained for elastin corrected for number of cells present (DAPI stain) as determined by ImageJ analysis and expressed relative to corresponding WT mice. The dashed line represents the average elastin levels in WT mice and error bars represent the standard deviation from the mean. ^*^^*^^*^p < 0.001. Black scale bars represent 10 μm (panels a–d). White scale bars represent 75 μm (panels e–h) and 10 μm (panels i–l).

Lamin A/C is a significant regulator of cell stability and dynamics (30), and its expression correlates with the stiffness of tissue (31). Increased rigidity of cardiomyocytes enhances passive tension *in vitro* and *in vivo*, resulting in functional heart defects (32,33). To gain insights into the wider impact of increased lamin A/C levels on the contractile apparatus of cardiomyocytes and overall stiffness of heart tissues in the *Smn^2B/−^* SMA mouse model, we examined the levels of elastin, a protein essential for the elastic properties of many tissues. Elastin was identified by iTRAQ analysis to be significantly reduced to 0.01-fold (*p* = 0.003) in heart tissue from the *Smn^2B/−^* SMA mouse model compared with WT. This finding was confirmed qualitatively by immunohistochemical analysis of van Gieson staining (i.e. a modified Miller’s stain which differentially stains elastin from collagen and muscle), and quantitatively by higher-power immunofluorescence analysis (0.30 ± 0.14-fold (*p* < 0.00001) in *Smn^2B/−^* vs WT) (Fig. 5). Elastin levels in *Smn^2B/−^* mice following treatment with either AAV9*_Co-hSMN1* or scAAV9*-SMN1* remained comparable to those observed in untreated *Smn^2B/−^* mice (0.37 ± 0.16-fold (*p* = 0.41), and 0.30 ± 0.13-fold (*p* = 0.24)) of WT levels respectively) (Fig. 5).

## Discussion

Over the last two decades, evidence has built to suggest that in severe forms of SMA, structural and functional abnormalities in heart tissues exist (14,18,22), whereas in patients with less severe types, a potentially milder impairment of cardiac function is suggested (9,16). The aim of this study was to conduct a proteomic comparison of heart tissues from the less-severe *Smn^2B/−^* SMA mouse model and age-matched WT mice and compare these findings with those identified in our previous study of heart tissues from the severe Taiwanese SMA mouse model (15) to determine whether a conserved response to reduced SMN levels is evident. We also investigated whether dysregulated proteins with a known role in heart function are impacted and whether their levels could be rescued by increasing SMN levels in the less-severe *Smn^2B/−^* mouse model.

In each SMA mouse model, hundreds of proteins were found to be dysregulated compared with age-matched WT mice. Bioinformatics analysis revealed a highly significant enrichment of terms relating to ‘abnormal morphology’ and ‘dilated cardiomyopathy’ in reference to ‘diseases & disorders, physiological system development & function’ in both dysregulated datasets. Previously, cardiac defects such as thinning of the interventricular septum and cardiomyocyte disorganization have been noted in the severe Taiwanese SMA mouse model (14) as have atrial or ventricular septal defects (11) and thickened myocardium (9) in patients with SMA type I. Out of 42 SMA type II and III patients, one patient had mitral valve prolapse, 7 had sinus tachycardia, and more than half had an elevated minimum 24-h heart rate (16). When ‘molecular and cellular functions’ were considered, terms relating to the ‘translation, synthesis and metabolism of proteins’, and ‘cell and organismal death’ were highly significant. Most of these terms are typically associated with SMA, in particular, SMN is known to be key for protein homeostasis (34). Both *in vitro* and *in vivo* studies provide support for impaired translation in SMA (35,36) and a comprehensive study involving rat primary neuron culture, fibroblasts from SMA Type I patients and neurons from a severe mouse model of SMA (*Smn^−/−^;SMN2^tg/0^*) demonstrated reduced *de novo* protein synthesis linked to mTOR signalling (37). In an earlier study, microfilament metabolism was found to be impacted by reduced levels of SMN (38), and with evidence building for a role of mitochondrial dysfunction in SMA (24,39–41), all these factors may contribute to altered protein metabolism. Most of the significantly enriched ‘canonical pathways’ common to both mouse models have also been implicated in SMA, such as EIF2 signaling (23), mitochondrial dysfunction (24) and mTOR signaling (37), but of particular interest was the significant enrichment of ‘dilated cardiomyopathy’ and the ‘dilated cardiomyopathy signaling pathway’ in both mouse models of SMA.

Although differentially abundant proteins from the *Smn^2B/−^* dataset were associated with several cellular components pertinent to cardiomyocyte function, the only cellular component to be significantly enriched among proteins common to both mouse models was intermediate filaments. Genetic mutations of the intermediate filaments lamin A/C and desmin are known contributors of cardiomyopathies and heart failure (25,42). Both proteins form networks within the cardiomyocyte and help maintain intra- and intercellular structure, with lamin A/C at the nuclear envelope (43) and desmin within the cytoplasm. In addition, lamin and desmin have important functions in nucleo-cytoskeletal coupling and mechano-transduction, gene regulation, metabolism, mitochondrial homeostasis and cardiomyocyte differentiation and survival (43–45), all of which are necessary for a fully functioning heart.

As previously confirmed in the Taiwanese mouse model of severe SMA (15), levels of lamin A/C were significantly increased in heart tissues from the *Smn^2B/−^* mouse model of less-severe SMA. Increased levels of lamin A/C were localized to the nuclear envelope and to the sarcomere of *Smn^2B/−^* mouse hearts, and further support the concept that appropriate lamin A/C production is SMN-dependent (15,27,46). Previously, increased lamin A/C levels have been described in muscle (47) and Schwann cells from SMA mice (48) and in motor neurons obtained from Type I SMA patients (49). In terms of impact on heart conditions and development of dilated cardiomyopathy, impairment of the lamin A/C gene is a well-known factor (50) and results in conduction problems, arrhythmias, atrioventricular block and sudden cardiac death (51–55) with at least 260 *LMNA* mutations having been linked to cardiac diseases (56). In several studies, *LMNA* mutations have also resulted in an SMA-like phenotype with cardiac involvement (26,27).

An increase in lamin A/C may result in a ‘stiffer’ nucleus which will alter the biomechanics of the cardiomyocyte, and thus the heart tissue, and may be in response to increased stiffness of the heart tissue (31). Conditions where increased accumulation of lamin A at the nuclear envelope is known to occur are Hutchinson-Gilford progeria syndrome and restrictive dermopathy. Both conditions arise from improper post-translational processing of prelamin A, the former from an *LMNA* mutation which results in a mutant farnesylated prelamin A (progerin) whilst the latter is due to reduced levels of ZMPSTE24, the enzyme necessary for processing prelamin A (57). Although not verified by immuno techniques in the current study, ZMPSTE24 was significantly increased in the mass spectrometry data from both SMA mouse models which may imply dysregulation of lamin A processing. In the current study, lamin A was also prevalent at the sarcomere, especially in *Smn^2B/−^* mice where it co-localized with alpha-actinin 2. This may indicate an increase in unprocessed lamin A in SMA, as previously prelamin A was identified at the sarcomere along with alpha-actinin 2 (55). As it has been suggested that prelamin A may be toxic and could promote dilated cardiomyopathy (55), further investigations into understanding whether lamin A processing is defective in SMA may prove useful. Additionally, the phosphorylation status of lamin A in SMA is worthy of investigation as this may have implications for the regulation of lamin A levels, as hypo-phosphorylation renders lamin A more stable and less likely to be degraded (58) and has occurred in mesenchymal stem cells when subjected to increased tissue stiffness (59).

Lamin A/C is the main provider of nuclear membrane mechanical strength and is key to transmitting mechanical force from the ECM to the nucleus (60,61). Deficiency or mutations in lamin A/C can cause mechano-transduction impairment (62) or impact whole-cell biomechanical properties (63). In a lamin A-deficient mouse model, the desmin network was disrupted and detached from the nuclear surface (64), suggesting defective force transmission due to loss of lamin interactions with desmin and subsequent loss of cytoskeletal tension. In another study of lamin A-deficient mice, desmin accumulation was increased in both muscle and cardiac tissues (65). It may prove useful to consider whether the reverse is true in SMA cardiomyocytes whereby decreased levels of desmin result in increased lamin A or *vice versa.*

Desmin is located throughout the cardiomyocyte and assists in transmitting force across the ECM, sarcomere and cytoskeleton (66), and has been implicated in SMA (67). In combination with microtubules and microfilaments, desmin helps to maintain the cytoskeleton structure and organization of sub-cellular organelles (68) and is thought to provide cardiomyocyte nuclei with tension (69). Structurally, desmin links individual myofibrils at the *Z*-discs (70,71), connects *Z*-discs to the intercalated disc (72), forms part of the desmosomes, and is linked to the costamere enabling adhesion to the ECM (73). Overall, desmin is key to the functioning of sarcomeres in efficient syncytia. Mutations often result in loss of sarcomere integrity and myofibrillar structure (74) and depletion causes muscle architecture disruption (75), which is not impacted during embryogenesis and myofibrillar assembly but affects muscle regeneration resulting in weaker mice in a desmin knock-out model (76). In other studies, lack of desmin impacted contractile function (77) and increased fibrosis (78) with muscle fibres lacking desmin being more susceptible to physical damage (79) and desmin loss causing increased passive stiffness in muscle (80).

Mechano-sensing within cardiomyocytes is a ‘two-way’ system, with the nucleus being an important mechanosensory organelle in the cell and intimately connected to the cytoskeleton, and the latter in turn to the ECM. For optimal heart performance, both electrical and mechanical stimuli are required with cardiomyocytes being subjected to cyclic contraction as the heart beats (81). Cardiomyocytes also experience passive mechanical stimuli such as that exerted by the cardiac ECM. The cardiac ECM is a combination of elastin bundles, collagen and interconnected basement membranes (82), with intra- and extracellular components contributing to overall myocardial stiffness (81). Although collagen is the main extracellular protein in myocardial tissue, elastin, which is found in the epicardium, endomysium and structures such as arteries, atria and ventricles of the heart (83), provides elasticity upon mechanical demand (84). Elastin is a structural glycoprotein and an essential component of myocardial stiffness (85), so any alterations in its levels will impact heart function. Typically, elastin is very stable with a considerably low turnover rate, with degradation or damage indicating pathological remodelling of the tissue and cardiovascular disease (86). Reduced levels of elastin, as found in this study, will increase the heart tissue’s passive stiffness, potentially reducing cardiac function, as cardiomyocytes cultured on a stiff, fibrotic-like matrix fail to beat properly (87). Typically, following myocardial infarction/ischaemia, elastin levels will decrease (88), promoting ECM remodelling, which further increases the stiffness of the cardiac tissue and impairment of cardiac function (85). In Williams-Beuren syndrome, impairment of the elastin *ELN* gene results in four out of five patients having cardiovascular abnormalities (89), with mutations also resulting in congenital heart disease (90). Elastin fibres are crucial for the proper functioning of Purkinje’s fibres (91) and blood vessels (92), and impairment can cause arrhythmias (93) or altered blood flow respectively, again impacting heart function.

Two AAV9-mediated *SMN1* treatments were used in this study, both of which have previously been used in *Smn^2B/−^* mice (28,29), but the novel codon-optimized cDNA transgene demonstrated significantly enhanced expression of the human *SMN1* gene in *Smn^2B/−^* mice hearts. Both treatments restored desmin levels to that of WT mice, but lamin A/C levels remained elevated and elastin levels decreased even though the codon-optimized cDNA transgene generated SMN levels more than 10-fold greater than those found in WT mice. The lack of effect of increased SMN levels on lamin A in this study may be due to several reasons: (i) lamin A is independent of SMN levels; (ii) the impact of low SMN levels on lamin A is greatest during embryonic development so post-natal treatment is too late; (iii) cardiac remodelling is influencing lamin A levels; and/or (iv) dysregulation of proteins responsible for post-translation processing or regulating the stability of lamin A are not independent of SMN levels. In contrast, desmin levels were increased by both AAV9-mediated *SMN1* treatments, indicating a direct relationship between these two proteins. Previously, abnormalities in the *Z*-bands of a patient with SMA were identified (94). As low levels of desmin appeared to be associated with sarcomere damage in a rat model of diabetes mellitus (95) and alpha-actinin staining showed disrupted sarcomere/costamere/*z*-disc striations in desmin-null mice (96), it is coherent that the improved levels of desmin in the AAV9-treated *Smn^2B/−^* mice result in improved sarcomere structure, although higher resolution microscopy is required to confirm this. Interestingly, SMN has been found to be localized to the I- and M-bands of sarcomeres in normal human skeletal fibres (97) and the *Z*-disc in mice (20) which suggests that sarcomeric SMN may directly or indirectly interact with the cytoskeleton and thus be involved in sarcomere structure (97), or its maintenance (20). In addition, during heart development, desmin and lamin A are abundant in the cardiac conduction systems; desmin preferentially in the myocardium of the central conduction system and lamin A more localized to the peripheral conduction system, then progressively to the central conduction system (98). It may be possible that AAV9-mediated treatment does not rectify lamin A/C levels due to inherent problems with the central conduction system that are established prior to treatment being administered. Additionally, abnormalities in conduction usually occur before dilated cardiomyopathy development (99) with elevated heart rate being associated with cardiomyopathy in late adolescence and increased heart failure risk (100). In desmin mutant mice, the distribution of lamin A/C was nucleoplasmic, whereas in WT mice, lamin A/C was mostly at the nuclear periphery (72). As AAV9-mediated treatment restored desmin levels, lamin A/C’s nuclear distribution may improve over time.

Other researchers have found the outcome of AAV9 treatment in SMA to be variable. A study focussed on fibrosis, remodelling, vascular integrity and oxidative stress in two mouse models of differing severity only found a partial rescue of cardiovascular defects in their mouse model of severe SMA and although structural defects were rectified, heart function incorporating heart rate, stroke volume and cardiac output remained significantly lower than WT mice, as did lifespan (13). In the SMNΔ7 mouse model, scAAV9 treatment resolved the bradycardia issues, but ‘cardiac output’ remained similar to untreated mice, probably as a result of ‘stroke volume’ remaining decreased following scAAV9 treatment (12). In addition, contractility remained lower than WTs following treatment. Another study using the SMNΔ7 SMA mouse model used the histone deacetylase inhibitor, trichostatin A, which is known to improve the phenotype of SMA mice. Although heart function improved, these mice did not survive as well as the WT mice (101). Typically, levels of lamin A/C in mouse cardiomyocytes, desmin in rat heart tissues and the elastin component of connective tissues decline with age (102–104), although in male F344/BN hybrid rats, desmin levels were found to increase in aging skeletal muscle (105). In future studies, it will therefore be important to characterize the temporal regulation of the proteins investigated throughout the disease time-course in the *Smn^2B/−^* mouse model, with and without AAV-9 mediated *SMN1* replacement, and to establish whether restoring levels of these proteins would have an impact on cardiac function of the mouse model. SMN may be essential for heart development prior to birth and thus scAAV9 treatment may not correct key development milestones involving SMN. Additionally, other factors such as lung and metabolic function or scoliosis may contribute to continued impairment of heart function in SMA. Prior to the introduction of treatments, such as nusinersen and risdiplam, which modify *SMN2* gene regulation to increase functional SMN expression, the impact on concomitant health issues in SMA patients following respiratory assistance, and thus extended life expectancy, has been previously highlighted (10) and included development of heart arrhythmia (bradycardia).

In conclusion, accumulating evidence suggests that cardiac assessment in the long-term care of SMA patients is warranted. We only investigated three of the dysregulated proteins identified from mass spectrometry analysis in the *Smn^2B/−^* mouse model, but there are many more that warrant further investigation, especially those pertinent to heart function. AAV9-*SMN1* gene therapy only corrected one of the proteins investigated, and although severe symptoms may be rare in SMA, subtle differences in cardiac performance in combination with current treatments increasing longevity in these patients, may impact cardiac health in future years. Thus, additional cardiac therapies may prove useful in optimizing treatment regimens for SMA patients.

## Materials and Methods

### SMA mouse models

The *Smn^2B/−^* and Taiwanese (original strain from Jackson Laboratories, No. 005058) mouse models were housed at Keele and Edinburgh Universities, respectively. As previously described (106), the less-severe *Smn^2B/−^* mouse model is maintained on a pure C57BL/6 genetic background with creation of the 2B mutation via a 3-nucleotide substitution within the exon splicing enhancer of exon 7 (107,108). The severe Taiwanese mouse model is heterozygous for the *SMN2* transgene on a *Smn* null background (*Smn−/−;SMN2^tg/+^*) (109). Tissues were harvested at a symptomatic time point of post-natal day (P) 18 and P8 for the *Smn^2B/−^* and Taiwanese models, respectively, in addition to age-matched WT mice. All experimental procedures on Taiwanese mice were authorized and approved by the University of Edinburgh Animal Welfare Ethical Review Body (AWERB) and UK Home Office (Project Licence 60/4569) in accordance with the Animals (Scientific Procedures) Act 1986. For the *Smn^2B/−^* mice, experimental procedures were authorized and approved by the Keele University AWERB and UK Home Office (Project Licence P99AB3B95) in accordance with the Animals (Scientific Procedures) Act 1986.

### Protein extraction and preparation for quantitative proteomic analysis

Total protein extracts were prepared as previously described (110) from the hearts of P18 *Smn^2B/−^* and age-matched WT animals (both *n* = 3) and isobaric tags for relative and absolute quantitation (iTRAQ) quantitative mass spectrometry analysis were added. Briefly, proteins were digested with trypsin, then peptides tagged with iTRAQ™ reagents as described in the iTRAQ kit protocol and sample groups assigned as follows: 116-P18 WT and 117-P18 *Smn^2B/−^.*

### High pH reverse-phase liquid chromatography fractionation and liquid chromatography–tandem mass spectrometry analysis

Fractionation and mass spectrometry analysis were carried out as previously described (15). In brief, the iTRAQ-labelled peptides were combined, concentrated, resuspended in buffer A (10 mm ammonium formate [NH_4_HCO_2_], 2% acetonitrile [MeCN], pH 10.0) then fractionated using high pH reverse-phase liquid chromatography (C18 column). Following rinsing of the column with 96% buffer A until the optical density returned to baseline, a gradient was run from 4 to 28% of buffer B (10 mm NH_4_HCO_2_, 90% MeCN, pH 10.0) for 30 min followed by another from 28 to 50% buffer B for 6 min. Buffer B at 80% was used to rinse the column for 5 min and the column then re-equilibrated with 4% buffer B for 11 min. Fractions (0.5 mL) were collected every 30 s and their UV chromatograms analysed. Fractions with similar peptide concentrations were combined resulting in 12 fractions which were vacuum dried then resuspended in 30 μL of 0.1% formic acid.

For the mass spectrometry analysis, one-third of each fraction was analysed by mass spectrometry. Following the separation of the peptides via liquid chromatography, peptides were loaded with buffer A (2% MeCN, 0.05% TFA in ultrapure water) and bound to an Acclaim PepMap100 AQ6 trap (100 μm × 2 cm) (Thermo Fisher Scientific), which was then washed for 10 min with buffer A. The analytical solvent system consisted of buffer A and buffer B (98% MeCN, 0.1% FA in ultrapure water) at a 300 nl/min flow rate. To elute the peptides the following gradient was utilized: linear 2–20% of buffer B over 90 min, linear 20–40% of buffer B for 30 min, linear 40–98% of buffer B for 10 min, isocratic 98% of buffer B for 5 min, linear 98–2% of buffer B for 2.5 min and isocratic 2% buffer B for 12.5 min. Eluent was sprayed with a NANOSpray II source (electrospray ionization) into the TripleTOF 5600+ tandem mass spectrometer (AB Sciex) under the control of Analyst® TF software (AB Sciex). The mass spectrometer was operated as previously described (15) and the raw mass spectrometry data files analyzed by ProteinPilot software, version 5.0.1.0 (Applied Biosystems) in addition to the Paragon™ database search and Pro Group™ algorithm using the UniProtKB/Swiss-Prot FASTA database. Paragon search analysis parameters for sample were: type ‘iTRAQ4plex (Peptide Labeled),’ cysteine alkylation ‘MMTS,’ digestion ‘trypsin’, instrument ‘TripleTOF’ and species ‘Mouse’ with processing parameters described as ‘quantitative,’ ‘bias correction,’ ‘background correction,’ ‘thorough ID’ and ‘biological modifications.’ A protein threshold of > 5 was used in the Pro Group algorithm to calculate the relative protein expression with the generation of an error factor and *p*-value, and Proteomics System Performance Evaluation Pipeline used to determine false discovery rate. The mass spectrometry proteomic data can be accessed at the following DOI: 10.6084/m9.figshare.23256374.

### Bioinformatics analysis

Data that met the criteria for differential expression in the *Smn^2B/−^* mice were analysed using DAVID Functional Annotation Bioinformatics 2021 (111,112) and IPA software (QIAGEN Inc., https://www.qiagenbioinformatics.com/products/ingenuity-pathway-analysis) (113). Proteins were deemed differentially abundant in the *Smn^2B/−^* mice in comparison to age-matched WT mice after the following filtering criteria were applied and proteins removed if; (i) they were identified from just one peptide, (ii) had expression changes of less than 25% (SMA vs WT) or (iii) had *p*-values of > 0.05 assigned to their expression changes. Only terms with at least three annotated proteins and a *p*-value of ≤ 0.05 were noted for the DAVID analysis.

### SMN replacement in *Smn^2B/−^* mice via viral vector treatment

For SMN replacement, AAV9 vectors were used from two sources, RY (single stranded AAV9*_eGFP and* AAV9*_Co-hSMN1*) (28) and BS (self-complementary scAAV9*-GFP* and scAAV9*-SMN1*) (29) and administered through the facial vein at P0 (5E10 vg/pup-7E10 vg/pup). The vector sourced from RY uses a codon-optimized cDNA transgene which has been developed to enhance levels of the functional SMN protein produced both *in vitro* and *in vivo* (28) whilst the vector from BS used standard cloning procedures as previously described (114). Following CO_2_ anaesthesia and exsanguination, heart tissues were harvested from the following groups at the symptomatic time-point of P18: untreated WT mice (*n* = 4), untreated *Smn^2B/−^* mice (*n* = 6), *Smn^2B/−^* mice treated with the vector plus GFP (RY, *n* = 5; BS, *n* = 4) and *Smn^2B/−^* mice treated with the vector plus *SMN1* (RY, *n* = 5; BS, *n* = 5).

### Protein extraction and western blotting

All extraction steps were carried out on ice and as previously described (115). Heart tissues were diced then homogenized with pellet pestles in 2× modified RIPA buffer (2% NP-40, 0.5% Deoxycholic acid, 2 mM EDTA, 300 mM NaCl and 100 mM Tris–HCl (pH 7.4)) plus protease inhibitor cocktail at 1:100 (Sigma-Aldrich; P8340), left on ice for 5 min then sonicated briefly at 5 microns for 10 s. This process was repeated an additional two times. The samples were centrifugated at 13 000 RPM (MSE, Heathfield, UK; MSB010.CX2.5 Micro Centaur) for 5 min at 4°C and protein concentration determined via the BCA protein assay (Pierce™, 23 227). The concentration of the protein extracts was adjusted to approximately 2 mg/ml, and they were heated in 2× SDS sample buffer (4% SDS, 10% 2-mercaptoethanol, 20% glycerol, 0.125 M Tris–HCl (pH 6.8) and bromophenol blue) for 3 min at 95°C prior to loading onto 4–12% Bis-Tris polyacrylamide gels (Life Technologies, Warrington, UK) for SDS-PAGE (Biorad, Hercules, CA, USA). A horizontal slice of the gel was excised and stained with Coomassie blue as an internal loading control for total protein, if necessary, protein concentrations were further adjusted to ensure even loading of the samples. The proteins in the remaining gel were transferred overnight via western blot onto a nitrocellulose membrane. Following blocking with 4% powdered milk, the membranes were incubated with primary monoclonal antibodies against: mouse anti-SMN (MANSMA12 2E6; 1:100 (116)); rabbit anti-lamin A/C (Abcam; ab169532; 1:2000); rabbit anti-desmin (Abcam; ab227651; 1:1000) in dilution buffer (1% FBS, 1% horse serum (HS), 0.1% bovine serum albumin (BSA) in PBS with 0.05% Triton X-100) for 2 h at RT or O/N at 4°C. Membranes were then incubated with HRP-labelled rabbit anti-mouse Ig (DAKO, P0260) or goat anti-rabbit Ig (DAKO, P0488) at 0.25 ng/ml in dilution buffer for 1 h followed by either West Pico or West Femto (both ThermoFisher) and bands imaged with the ChemiDoc™ Touch Imaging System (Bio-Rad). Using ImageJ Fiji software (v1.51) (117) densitometry measurements of both antibody reactive bands and Coomassie-stained gel bands were undertaken.

### Immunofluorescence microscopy

Following excision, heart tissues incorporating the left and right ventricles were flash frozen in liquid nitrogen and stored at −80 °C then sectioned (7 μm) on a rotary cryostat, collected onto polylysine-coated slides and stored at −20 °C. Prior to staining, slides were brought to RT. All subsequent steps were carried out at RT and PBS used for each wash step (3 × 5 min) whilst blocking buffer (1% FBS, 1% HS, 0.1% BSA in PBS) was used for antibody dilution. Sections were washed, then blocked for 30 min prior to being incubated either for 2 h at RT or O/N at 4°C with primary antibodies against mouse anti-SMN (MANSMA12 2E6; 1:4 (116)), rabbit polyclonal anti-lamin A/C (NOVUS; NBP2-19324; 1:100), rabbit monoclonal anti-desmin (Abcam; ab227651; 1:100), rabbit polyclonal anti-elastin (Fisher Scientific; PA5-99418; 1:100) or mouse anti-alpha Actinin 2 (Gene Tex; GTX632361; 1:200). Following washing, 5 μg/ml of secondary antibody (Molecular Probes; goat anti-rabbit IgG ALEXA Flour 488; A11034) was applied for 1 h. Sections were washed prior to being stained with 4′,6-diamidino-2-phenylindole (DAPI; D9542; 0.4 μg/mL) for 10 min, then washed, mounted and imaged with a Leica TCS SP5 spectral confocal microscope (Leica Microsystems, Milton Keynes, UK).

Images generated were analyzed via ImageJ. For lamin A, images obtained with the 63x objective with an additional 4-fold magnification were utilized, whereas images taken with the 63× objective were used for desmin and elastin analysis. For the lamin A sections, a minimum of 100 cells were analyzed whereas for desmin and elastin a minimum of 200 cells were analyzed per condition. For each analysis the scale function was removed, so only the pixels either staining for DAPI or the protein (lamin A, desmin and elastin) were counted. Using the threshold function (118), the highlighted pixels were analyzed, and protein staining was corrected for cells present by dividing by the measure obtained for the positively stained DAPI areas. The fold difference between the SMA mouse models and their relevant WTs were then determined.

### Histochemical staining of elastin

Heart tissue sections were also stained for elastin with the Miller’s Elastic Van Gieson Stain Kit (Atom Scientific Ltd; RRSK11-100) using the protocol provided. All wash steps were carried out with tap water unless otherwise stated. Briefly, sections were washed then treated with acidified potassium permanganate (0.5%) for 5 min. Following further washing the sections were bleached with oxalic acid (1%) for 1 min, washed, then rinsed in 95% alcohol and submerged in Miller’s elastin stain for 3 h at RT. Sections were then rinsed with 95% alcohol until the slides were clear, washed, and counterstained with filtered Van Gieson stain for 2 min, then blotted, dehydrated, cleared and mounted.

### Statistical analysis

Statistical analyses were carried out using GraphPad Prism version 9.0.0 for Windows, GraphPad Software, San Diego, California USA, www.graphpad.com. Data distribution was assessed with the Shapiro–Wilk test and the unpaired two-tailed *t*-test and Mann–Whitney *U* test applied to parametric and non-parametric data, respectively. Western blot densitometry measurements were assessed using an unpaired, one- or two-tailed *t*-test with Welch’s correction (two-sample equal variance), depending on the null-hypothesis that was tested in each case. Venn diagrams were generated via a web-based tool (119). Data are presented as mean ± standard deviation with a *p*-value of ≤ 0.05 being considered as significant for all analyses.

## Supplementary Material

Supplementary_File_ddad121

Supplementary_Tables_ddad121

## Data Availability

The mass spectrometry proteomic data can be accessed at the following DOI: 10.6084/m9.figshare.23256374
