# Supplementary material for: AAV9-mediated SMN gene therapy rescues cardiac desmin but not lamin A/C and elastin dysregulation in Smn2B/− spinal muscular atrophy mice
Source: Hum Mol Genet. 2023 Jul 27;32(20):2950–65. doi: 10.1093/hmg/ddad121 (PMC10549791; doi:10.1093/hmg/ddad121)
Supplement: Supplementary_File_ddad121 [file supplementary_file_ddad121.docx]

**AAV9-mediated *SMN* gene therapy rescues cardiac desmin but not lamin A/C and elastin dysregulation in *Smn^2B/-^* spinal muscular atrophy mice**

**Supplementary File**

Sharon J Brown^a,b^, Darija Šoltić^a,b^, Silvia A Synowsky^c^, Sally L Shirran^c^, Ellie Chilcott^d^, Hannah K Shorrock^e^, Thomas H Gillingwater^e^, Rafael J. Yáñez-Muñoz^d^, Bernard Schneider^f,g^, Melissa Bowerman^b,h^ and Heidi R Fuller^a,b^*

Sharon J Browna,b, Darija Šoltića,b, Silvia A Synowskyc, Sally L Shirranc, Ellie Chilcottd, Hannah K Shorrocke, Thomas H Gillingwatere, Rafael J. Yáñez-Muñozd, Bernard Schneiderf,g, Melissa Bowermanb,h and Heidi R Fullera,b*

^a^School of Pharmacy and Bioengineering, Keele University, ST5 5BG, UK; ^b^Wolfson Centre for Inherited Neuromuscular Disease, TORCH Building, RJAH Orthopaedic Hospital, Oswestry SY10 7AG, UK; ^c^BSRC Mass Spectrometry and Proteomics Facility, University of St Andrews, St Andrews KY16 9ST, UK; ^d^AGCTlab.org, Centre of Gene and Cell Therapy, Centre for Biomedical Sciences, Department of Biological Sciences, School of Life Sciences and the Environment, Royal Holloway University of London, Egham Hill, Egham, Surrey TW20 0EX, UK; ^e^Edinburgh Medical School: Biomedical Sciences, University of Edinburgh, UK; Euan MacDonald Centre for Motor Neurone Disease Research, University of Edinburgh, Edinburgh EH8 9XD, UK; ^f^Bertarelli Platform for Gene Therapy, Ecole Polytechnique Fédérale de Lausanne (EPFL), Geneva, Switzerland; ^g^Brain Mind Institute, Ecole Polytechnique Fédérale de Lausanne (EPFL), 1015 Lausanne, Switzerland; ^h^School of Medicine, Keele University, ST5 5BG, UK

*Corresponding author

Email: [h.r.fuller@keele.ac.uk](mailto:h.r.fuller@keele.ac.uk)

Postal Address:

Telephone: +44(0)1782734546


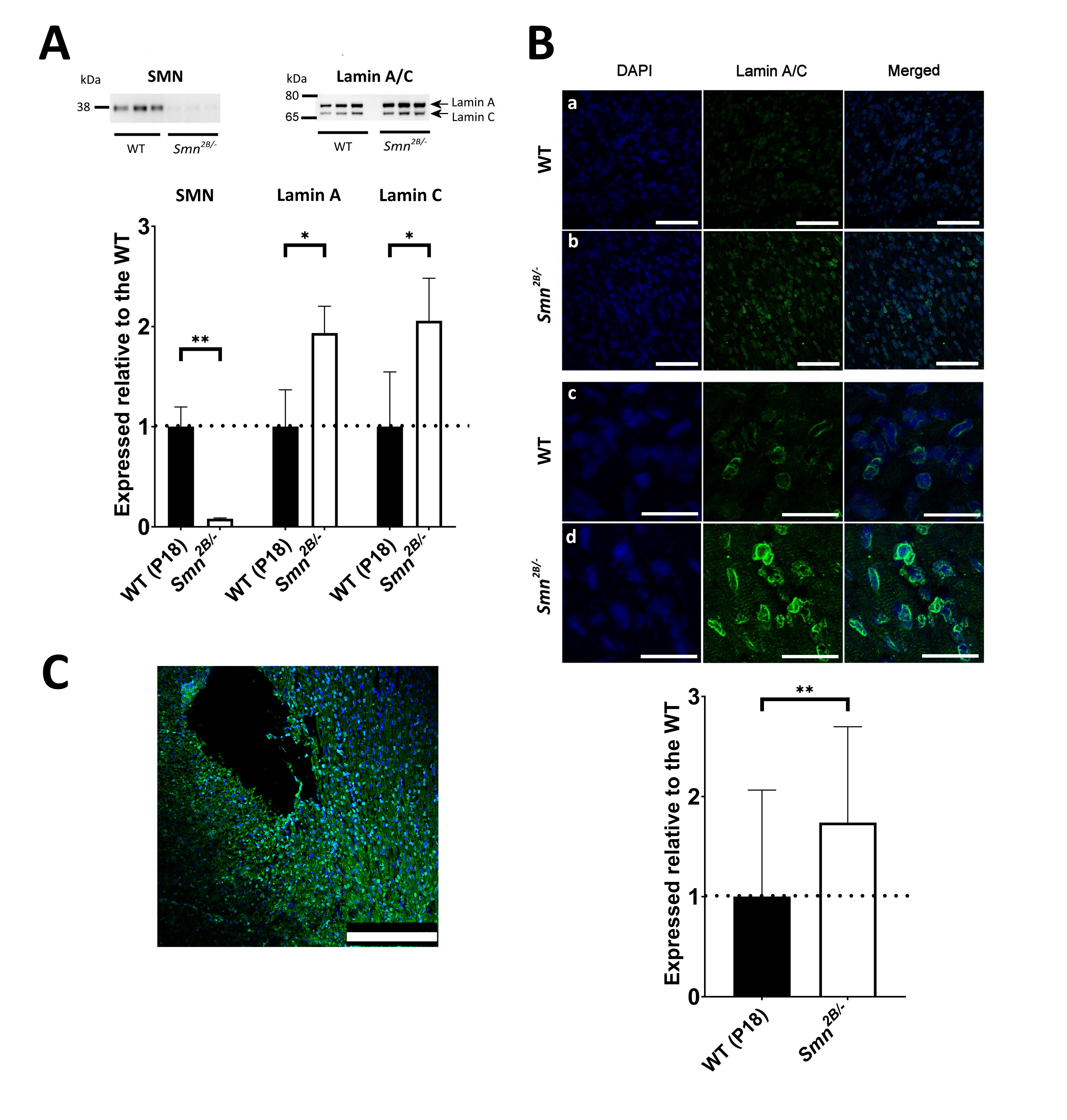


**Supplementary Figure 1: Levels of SMN and lamin A/C in heart tissues from the *Smn^2B/-^* mouse model of SMA.** **(A)** Representative western blots of SMN and lamin A/C levels in heart tissues from the *Smn^2B/-^* mouse model and age-matched WT mice (P18). The bar graph represents average SMN and lamin A/C protein levels expressed relative to WT mice. **(B)** Representative IMFs for lamin A staining within heart tissues from the *Smn^2B/-^* mouse model and age-matched WT mice with the bar graph reflecting area of cells stained for lamin A corrected for number of cells present (DAPI stain) as determined by ImageJ analysis and expressed relative to WT mice. Panels a, b: low magnification; panels c, d: high magnification. **(C)** Low-magnitude image of heart tissue to demonstrate that lamin A staining is not picking up blood cells within heart lacunae. Dashed line represents the average protein levels in WT mice and error bars represent the standard deviation from the mean. * *p*<0.05; ***p*<0.01. Scale bars represent 75 µm (B: panels a & b); 25 µm (B: panels c & d); 250 µm (C).


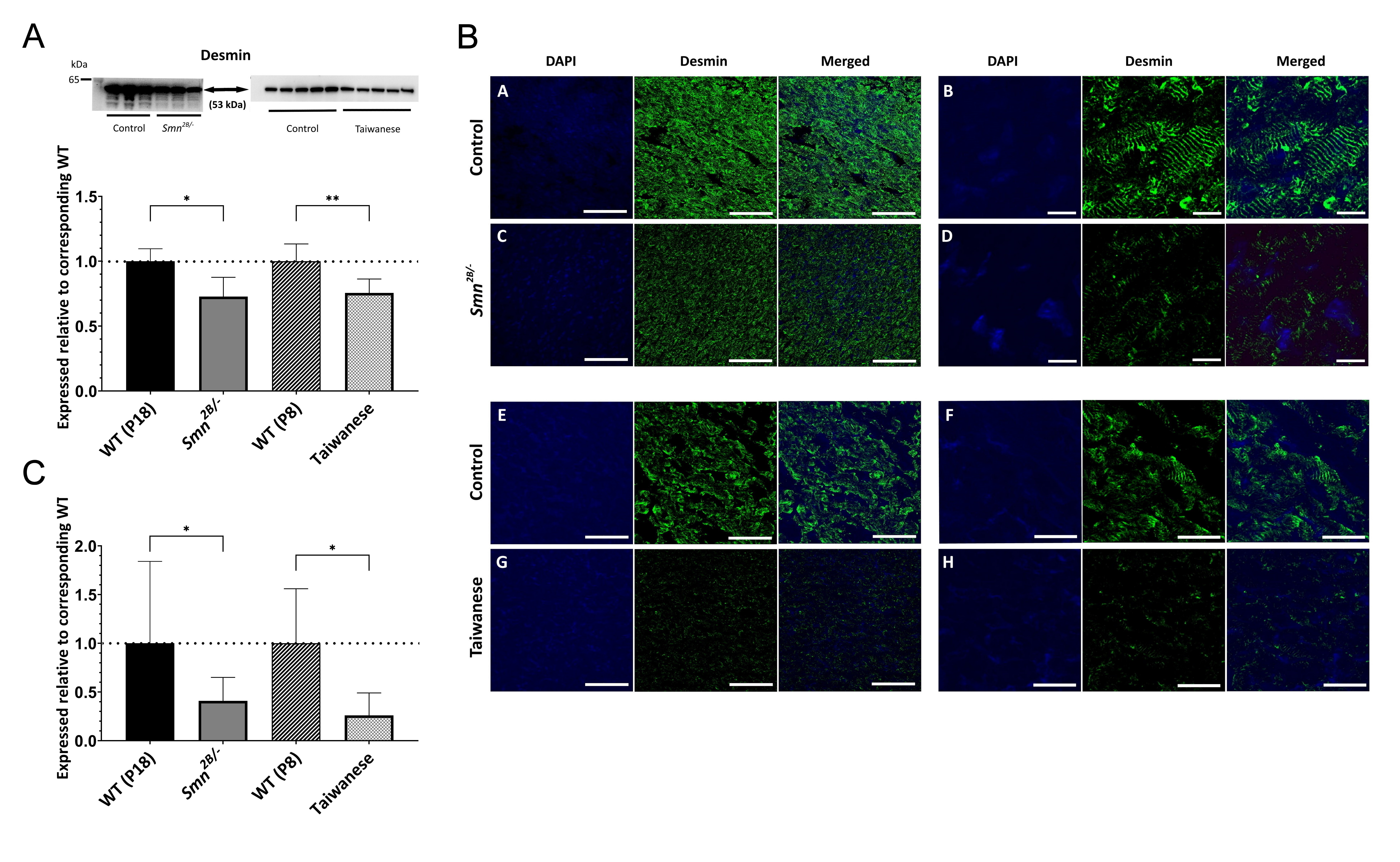
**Supplementary Figure 2: Levels of desmin in the heart tissues from the *Smn^2B/-^* and Taiwanese mouse models of SMA.** **(A)** Representative western blots of desmin levels in heart tissues from the *Smn^2B/-^* and Taiwanese mouse models of SMA and their corresponding age-matched WT mice (P18 & P8 respectively). The bar graph represents average desmin levels expressed relative to corresponding WT mice. **(B)** Representative IMFs for desmin staining within heart tissues from *Smn^2B/-^* and Taiwanese mouse models of SMA and age-matched WT mice, shown at two different magnifications for each model. **(C)** Bar graph reflecting area of cells stained for desmin corrected for number of cells present (DAPI stain) as determined by ImageJ analysis and expressed relative to corresponding WT mice. Dashed line represents the average desmin levels in WT mice and error bars represent the standard deviation from the mean. * *p*<0.05; ***p*<0.01. Scale bars represent 75 µm (B: panels A, C, E & G); 25 µm (B: panels F & H); 10 µm (B: panels B & D).
